# Efficacy of standardized training on a virtual reality simulator to advance knee and shoulder arthroscopic motor skills

**DOI:** 10.1186/s12891-018-2072-0

**Published:** 2018-05-16

**Authors:** Stefan Rahm, Karl Wieser, David E. Bauer, Felix WA Waibel, Dominik C. Meyer, Christian Gerber, Sandro F. Fucentese

**Affiliations:** Orthopaedic Department, Balgrist University Hospital, University of Zurich, Forchstrasse 340, CH-8008 Zurich, Switzerland

**Keywords:** Education, Simulation, Arthroscopy, Orthopaedic surgery, Virtual reality simulation, Training

## Abstract

**Background:**

Most studies demonstrated, that training on a virtual reality based arthroscopy simulator leads to an improvement of technical skills in orthopaedic surgery. However, how long and what kind of training is optimal for young residents is unknown. In this study we tested the efficacy of a standardized, competency based training protocol on a validated virtual reality based knee- and shoulder arthroscopy simulator.

**Methods:**

Twenty residents and five experts in arthroscopy were included. All participants performed a test including knee -and shoulder arthroscopy tasks on a virtual reality knee- and shoulder arthroscopy simulator. The residents had to complete a competency based training program. Thereafter, the previously completed test was retaken. We evaluated the metric data of the simulator using a z-score and the Arthroscopic Surgery Skill Evaluation Tool (ASSET) to assess training effects in residents and performance levels in experts.

**Results:**

The residents significantly improved from pre- to post training in the overall z-score: − 9.82 (range, − 20.35 to − 1.64) to − 2.61 (range, − 6.25 to 1.5); *p* < 0.001. The overall ASSET score improved from 55 (27 to 84) percent to 75 (48 to 92) percent; p < 0.001. The experts, however, achieved a significantly higher z-score in the shoulder tasks (*p* < 0.001 and a statistically insignificantly higher z-score in the knee tasks with a *p* = 0.921. The experts mean overall ASSET score (knee and shoulder) was significantly higher in the therapeutic tasks (*p* < 0.001) compared to the residents post training result.

**Conclusions:**

The use of a competency based simulator training with this specific device for 3-5 h is an effective tool to advance basic arthroscopic skills of resident in training from 0 to 5 years based on simulator measures and simulator based ASSET testing. Therefore, we conclude that this sort of training method appears useful to learn the handling of the camera, basic anatomy and the triangulation with instruments.

## Background

Training of residents is a very important, but demanding and time consuming job in teaching hospitals. Arthroscopic surgery in particular is difficult to learn since it cannot be acquired by observation and assisting alone [1, 2]. Virtual-reality based training has become more popular in the past, but there is still a lack of a standardized integration of virtual reality based simulator training in orthopaedic post-graduate programs [3–10]. The benefit of virtual-reality based teaching is proven to be at least of equal value as direct observation, animal and/or cadaver models or videotape learning tools [11–13].

The potential 24/7 availability of a training tool is increasingly important since the hands-on operation time gets less for residents. The initial high investment of such a simulator as well as the service expenses are recognized downsides. In a previous study [14] it was shown that medical students have a steep learning curve in the first 2 hours of training on a virtual reality based knee arthroscopy simulator but there were no identifiable predictors of talent or magnitude of improvement of skills.

It is not known which type of training on a virtual-reality based arthroscopy simulator is the most efficient and beneficial for residents. Further, there are different types of measuring the quality of an arthroscopic procedure and there is still a lack of validated outcome score for the evaluation of an arthroscopic performance [10]. Metric data such as task time or instrument movements can be measured and the skill level of an arthroscopist can be evaluated with scores such as the Arthroscopic Surgery Skill Evaluation Tool (ASSET) [5, 15].

It was the purpose of this study to determine whether a standardized, competency based training advances arthroscopic skills of residents. Two hypotheses were tested: First, arthroscopic novices (residents) improve their skills significantly through a standardized competency based training program on a virtual reality simulator. Second, the residents can reach an arthroscopic proficiency skill level based on the metric data and the ASSET score compared to fellowship trained arthroscopists after the training program.

## Methods

Prior to the study all participants gave their written, informed consent. Our local IRB waived the need for ethical approval; BASEC Nr: Req-2016-00442.

Twenty residents (16 males and 4 females) with a mean age of 31 (27 to 37) years and a mean of 2.5 (0 to 5.5) years experience in the orthopaedic field were included in this study. Furthermore, there were five fellowship trained experts (all males) with a mean age of 45 (34 to 63) years and a mean of 17 (8 to 35) years experience in the orthopaedic field also included in this study as a reference group. Three of them were shoulder specialists and two of them knee specialists, of which all had a very long experience in arthroscopic surgery. Detailed information on demographic data and previous experience in real and simulated arthroscopy are depicted in Table 1.

**Table 1:** Overview of the demographic information of the residents and the experts

Total arthroscopies performed	0	1 to 20	21 to 50	51 to 100	> 100	> 200
Residents	6	1	5	0	0	0
Experts	0	0	0	0	0	5
Total hours on knee / shoulder virtual reality based	0	1 to 5	6 to 20			
Residents	3	10	7			
Experts	1	3	1			
Total hours of playing video games per week	0	1 to 5	6 to 20			
Residents	19	1	0			
Experts	4	1	0			
Handedness	right	left				
Residents	16	4				
Experts	5	0				

The distribution of the demographic data and previous real arthroscopy- and simulator experience are depicted of the 20 residents and the five experts

### Protocol

All participants (residents and experts) performed a test on the validated VirtaMed AG (Schlieren, Switzerland) virtual reality based knee- and shoulder arthroscopy simulator [3, 16]. The residents had to pass a competency based training program, which included several knee- and shoulder arthroscopy tasks. Thereafter, the previously completed test was redone.

#### Test description

To start all participants, who did not know the simulator (*n* = 9) got a standardized introduction, explaining the hard- and software. A 2 minutes hands on time trying out the test tasks was allowed to all these participants. Further, all participants could get familar with the system using the camera and the tools for exactly 60 s.

#### Knee

Three knee arthroscopy tasks needed to be performed. One standard diagnostic knee arthroscopy task including the visualisation of the complete intraarticular anatomic structures. One foreign body removal task including fishing of six rings with the hook (three in the lateral and three in the medial compartment) and one guided task of removal of a flaptear of the lateral meniscus using the punch to achieve a stable meniscus. The time was stopped when the participants judged their job to be completed.

#### Shoulder

Twice, the same two shoulder arthroscopy tasks were performed, once in beach chair and once in lateral decubitus position. First, a standard diagnostic shoulder arthroscopy using the camera through the posterior portal visualizing the intraarticular and the subacromial (coracoacromial ligament and the acromioclavicular joint) structures was performed. Second, a therapeutic task consisting in touching five balls by the probe for 3 seconds. Either the anterior or anterolateral portal could be used.

#### Training protocol

The training protocol was, as mentioned before, competency based, which means, that residents with a certain level of performance regarding operation time and overall camera and instrument pathway were allowed to proceed to the next task and therefore have a shorter overall training period. Less experienced residents, which did not achieve a certain benchmark, had to repeat the tasks until it was completed in the required level. The training program was chosen to be at least 3 hours and maximum 5 hours if all tasks were repeated to the maximum. All participants began with training of knee arthroscopy. Overall 32 tasks including diagnostic and therapeutic arthroscopy were completed. The tasks comprised diagnostics, guided diagnostics, diagnostics and palpation with the probe, catching rings with the hook, foreign body removal with the grasper/hook (stars) and partial meniscectomy.

Afterwards the training continued with shoulder arthroscopy, first in beach chair and then in lateral decubitus position. Twenty tasks each including diagnostic and therapeutic arthroscopy were completed. The tasks comprised diagnostics, guided diagnostics, diagnostics and palpation with the probe, catching rings with the hook, foreign body removal with the grasp.

#### Outcome parameters

##### Metrics

All the metric data (time in seconds, camera, hook/probe and punch pathway in centimeters) were recorded by the simulator system. We evaluated these metric data of the simulator itself and additionally used z-scores for comparison of the tests [14].

##### Arthroscopic surgery skill evaluation tool (ASSET)

Furthermore, of two particular knee (one diagnostic arthroscopy/one therapeutic arthroscopy catching six rings) and two shoulder (one diagnostic arthroscopy in beach chair/one therapeutic arthroscopy palpating five balls with the probe in lateral decubitus) arthroscopy tasks, the ASSET was evaluated to measure the performance. The ASSET score is based on these following domains; safety, field of view, camera dexterity, instrument dexterity, bimanual dexterity, flow of procedure, quality of procedure, which had to be scored from one to five (one being a novice level and five an expert level). The additional domains, autonomy and added complexity of procedure were not evaluated in this study. This scoring was performed by two experienced knee and shoulder surgeons (S.R. and K.W.) in a fully blinded (pre- or post training/resident or expert) consensus read-out. As instrument and bimanual dexterity in diagnostic arthroscopies could not be evaluated, the maximum ASSET score was 25 points for the diagnostic tasks and 35 points for the therapeutic tasks, respectively.

The metric data were correlated to the ASSET score. The learning curve of the residents using the metric data and the ASSET scores were analyzed. The final post training test results of the residents were compared with the basic test results of the experts. Furthermore, the demographic data from the questionnaire were correlated with the metric data and the ASSET score of the final basic test.

### Statistical analysis

Data are presented as mean and standard deviation (SD) for continuous variables and as proportion (%) for categorical variables, if not stated otherwise. A two-tailed Kolmogorov–Smirnov test was used for testing normal distribution, if *p* ≤ 0.05, the data were considered as normally distributed. The Wilcoxon signed-rank Test was used for testing differences between means of pre- to post training results for each participant. The Mann-Whitney U Test was used for testing differences between the expert and non-expert subpopulations.

The Pearson’s Chi-squared test was applied for testing differences of distribution of categorical variables. A *p*-value of < 0.05 was considered to be statistically significant.

For comparison of metric data of different dimensions, variables were normalized to the expert population by subtracting the individual score of each participant from the expert populations mean and dividing the difference by the expert populations standard deviation. The thereby calculated z-scores of each variable were then summed up and an arithmetic mean was calculated to obtain a single score for each task. The statistical analysis was performed by a professional bio-statistician.

## Results

### Metrics

The overall mean z-score of the basic test improved significantly pre- to post training from − 9.82 (range, − 20.35 to − 1.64; SD 5.05) to − 2.61 (range, − 6.25 to 1.5; 1.63); *p* < 0.001. A statistically significant improvement was found in all pre- to post training metric shoulder tasks and all except two metric knee tasks. Only the hook pathway in the removal of six rings and the punch pathway in the partial meniscectomy task showed no significant difference from pre-to post training. (see Table 2).

**Table 2:** Overview of the metric pre- to posttraining results and the expert results

Test; Task	Metric Parameter	Residents Pretraining Test (mean)	min	max	SD	Residents Posttraining Test (mean)	min	max	SD	Wilcoxon: pre-to-posttraining	Experts Test (mean)	min	max	SD	Mann-Whitney-U: Residents posttraining to experts
Knee: Diagnostic Arthroscopy	Time (sec)	254	77	571	133	121	79	219	39	< 0.001	118	71	174	50	0.621
	Camera pathway (cm)	145	39	621	127	74	40	191	36	0.001	75	42	113	30	0.767
Knee: Removal of 6 rings	Time (sec)	159	77	367	83	97	55	203	40	0.001	65	53	73	8	0.029
	Camera pathway (cm)	50	18	174	42	32	12	79	19	0.048	20	14	27	5	0.169
	Hook pathway (cm)	107	38	343	76	82	27	350	74	0.067	53	39	84	19	0.243
Knee: Lateral partial meniscectomy	Time (sec)	332	128	952	235	149	109	231	35	< 0.001	134	87	191	50	0.408
	Camera pathway (cm)	147	53	665	159	70	35	200	35	0.001	87	47	135	35	0.243
	Punch pathway (cm)	42	20	77	16	33	17	60	12	0.057	46	22	77	25	0.447
Shoulder (BC): Diagnostic Arthroscopy	Time (sec)	439	201	728	189	186	91	425	79	< 0.001	60	31	95	25	< 0.001
	Camera pathway (cm)	627	152	3291	694	201	67	385	90	< 0.001	73	25	107	33	0.001
Shoulder (BC): Touching of 5 balls	Time (sec)	271	108	704	171	121	58	244	43	< 0.001	66	43	82	16	< 0.001
	Camera pathway (cm)	103	21	272	74	48	14	118	25	0.004	28	17	33	7	0.071
	Hook pathway (cm)	282	96	1018	233	101	55	286	54	< 0.001	59	33	80	21	0.035
Shoulder (LAT): Diagnostic Arthroscopy	Time (sec)	296	96	1026	213	151	77	399	72	0.005	70	51	99	18	< 0.001
	Camera pathway (cm)	288	79	1152	237	145	62	385	74	0.004	73	57	81	9	0.003
Shoulder (LAT): Touching of 5 balls	Time (sec)	155	50	693	156	61	30	117	21	0.003	42	31	57	11	0.042
	Camera pathway (cm)	93	20	444	116	38	16	66	15	0.023	21	15	27	5	0.015
	Hook pathway (cm)	182	53	505	122	72	27	144	36	0.005	33	28	38	5	0.003

Complete overview of the results. The metric data are all seperately depicted using the mean, maximum, minimum and standard deviation. The p-value was calculated using the Wilcoxon signed rank test

The experts reached an overall mean z-score of 0.0 (range, − 0.55 to 0.52; SD 0.47) which was significantly better than the post training result of the residents (*p* < 0.001). The experts reached a higher mean *knee z-score* with 0.00 (range, − 0.69 to 0.83; SD 0.61) than the post training residents result with − 0.87 (range, − 4.48 to 0.66; SD 1.50) not reaching statistical significance; *p* = 0.192.

In the the experts scored a significantly higher score with 0.00 (range, − 0.47 to 0.63; SD 0.46) compared to the post training z-score of the residents − 4.35 (range, − 8.56 to − 0.36; SD 2.28); *p* < 0.001. The results between the lateral decubitus and the beach chair position did not significantly differ.

All except one parameter in the knee tasks (time in the knee removal of six rings) showed no significant difference between the post training result and the expert result. However, in the shoulder tasks the experts were significantly better in all but one parameter (camera pathway in beach chair position of shoulder touching five balls). A complete overview of the pre- to post training results and the expert results of every parameter is depicted in Table 2.

### Asset

**Fig. 1:**
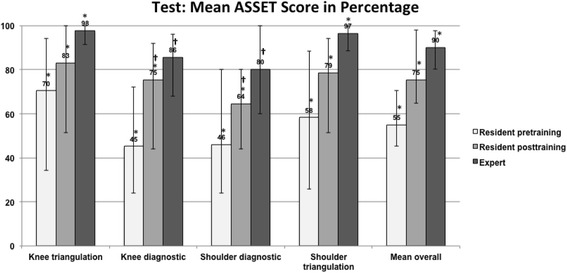
Shows the percentage of the mean ASSET score of the four tested tasks. It shows a significant improvement in all four tasks from pre- to post training in the residents. Additionally there was again an improvement from the expert group with a better result in the diagnostic tasks and a significantly better result in the therapeutic tasks of knee- and shoulder arthroscopy

The mean overall ASSET score improved significantly in all four tasks from pre- to post training; from 55% (range, 27 to 84) to 75% (range, 48 to 92); *p* = 0.001. In all four tasks the residents reached a higher (or close to reference) score in the post training assessment, although the experts ASSET score was still significantly better in two tasks (knee triangulation (rings) and shoulder triangulation (balls)). In Fig. 1 the complete results of the ASSET score are summarized.

### Correlations

There was a good correlation (correlation coefficient = 0.688; *p* = 0.013) between the overall z-score and the overall ASSET score. There were no positive or negative correlations of the data regarding previous real or simulated experience, the handedness, the sports activity and the video games experience to the improvement of the scores or the final score.

## Discussion

The most important finding in this study is that a standardized, competency based training leads to significant improvements of arthroscopic skills measured by both, metric data as well as structured assessment of performance and safety using the ASSET score. The difference between experts performance and residents post training remains significantly bigger for shoulder arthroscopy than for knee arthroscopy (see Fig. 1). Therefore, we could confirm our hypothesis that a significant improvement is achieved for residents, but had to reject the second hypothesis that a proficiency level, similar to the one of a fellowship trained arthroscopist, based on the metric data and the ASSET score can be reached by simulator training alone.

The significant improvement from pre- to post training was shown in the objective metric simulator data (overall z-score, *p* < 0.001, overall ASSET score *p* < 0.001). However, the residents improved their knee arthroscopy skills to a level almost as good as the experts, not showing a significant difference in the z-scores; *p* = 0.192. There was no correlation between the previous experience in arthroscopy and/or simulator training (especially knee arthroscopy) to the tests. On the other hand 50% of the residents already had knee arthroscopy simulator experience compared to only 30% having had shoulder. The tests in the simulated shoulder arthroscopy might have been less anatomically defined, favouring actual surgical experience and therefore explaining the somewhat smaller effect.

The clear correlation of the metric data and the ASSET score, used to measure the arthroscopic skill level including aspects such as the flow of the camera and instruments, as well as the safety aspect, such as not scratching the intraarticular cartilage, is another important finding in this study. Good final ASSET scores of the residents in the diagnostic tasks (shoulder and knee), which were not significantly inferior to the experts, was a confirmation of our tested training program. Also, for therapeutic arthroscopy, where an additional instrument was used and triangulation required, a strong increase for the residents was seen particularly in the knee. It seems that this competency based approach works and all participants improved relevantly with a variable time, which has been spent on the simulator (fast learners vs slow learners).

We see the main value of the method in improved camera handling relative to the anatomy to be identified, as well as in the improvement of triangulation skills. For this purpose we intend to use this training program on a regular basis in the future. In addition a relevant advantage of this type of training is the independency from additional medical staff. Other practical arthroscopic issues, such as swelling of the tissue and bleeding can of course not be fully simulated with the current technology. Nevertheless, from a handling standpoint, we feel that residents with a completed simulator training have a competent skill basis for a step-wise initiation of their OR surgical training and confirm similar investigational results for the shoulder (Waterman et al. [17]) or for laparoscopic surgery (Aggarwal et al. [1]) To define a benchmark (Angelo et al. [18]) at what level real expert level is reached, remains difficult, particularly considering that the best results of the trained residents exceeded those of some experts. When looking at maximally reached values however, the best expert was usually still approximately twice as fast as the best resident, indicating an actual correlation with practical experience. Our findings are in line with a finding of Ferguson et al. who stated that basic arthroscopic skills can not immediately be transferred to an unfamiliar anatomical environment within a simulated setting [19]. In a study of Jackson et al. there is evidence, that residents can retain their skills over a time period of 6 months [20]. Our study did not test this important point in skill retaining.

As a limitation, the study group showed a natural, but large heterogeneity for the residents in all different stages with different levels and experience in real arthroscopy and on the simulator, which may explain the poor correlations with the demographic data. Still there were significant improvements found regardless the previous experience. The intensity of the training was not standardized by number of hours spend on the simulator, but we have designed a proficiency based curriculum consisting of 32 knee arthroscopy and 2 × 20 shoulder arthroscopy cases (to be completed in both, beach chair and lateral position). Participants had to repeat the cases until they passed a pre-defined performance level. Using this approach, we were able to have shorten the training for fast learners and for participants with higher baseline performance. On average, training time was in the range of 3-5 h (which is in accord with reports) [14]. A limitation of the current study is the fact, that the training frequency was not standardized: some of the participants completed baseline assessment, training and post-assessment on 1 day. We observed that in some of these participants, the learning effects were covered by fatigue effects resulting in “negative learning effects”, i.e. some participants showed lower performance after training than at baseline. As we know that more training would add some further skill improvement [21], the average training effects reported in this study are smaller than they would be in reality. Reppenhagen et al. systematically analysed optimal training frequency for arthroscopic skill acquisition and concluded that a training frequency of two sessions per week leads to best skill acquisition and retention [22].

The important question remains whether these results can be transferred in the operating room. Considering the improvement in basic arthroscopic triangulation skills, reaching for some residents the level of experts in the simulator, our results seem to support earlier findings regarding the positive transfer validity in orthopaedic surgery [21, 23]. To lay a basis for the handling of the camera and instruments we conclude that this sort of training makes sense and should be integrated in residents training programs since the hands on time in the operating room decreases for current residents in training [24].

## Conclusions

The use of programmed simulator training with this specific device for 3-5 h is an effective tool to advance basic motor skills of residents in training from 0 to 5 years based on simulator measures and simulator based ASSET testing. Therefore, we conclude that this sort of training method appears useful to learn the handling of the camera, basic anatomy and the triangulation with instruments.
